# *ANO6* (TMEM16F) inhibits gastrointestinal stromal tumor growth and induces ferroptosis

**DOI:** 10.1515/med-2024-0941

**Published:** 2024-05-15

**Authors:** Hao Wang, Wei Zhao, Daorong Wang, Jin Chen

**Affiliations:** School of Public Health, Nanjing Medical University, Nanjing 211166, China; Department of Gastrointestinal Surgery, The Yangzhou School of Clinical Medicine of Nanjing Medical University, Yangzhou 225001, China; General Surgery Institute of Yangzhou, Yangzhou University, Yangzhou 225001, China; Yangzhou Key Laboratory of Basic and Clinical Transformation of Digestive and Metabolic Diseases, Yangzhou 225001, China; The Key Laboratory of Modern Toxicology, Ministry of Education, Center for Global Health, School of Public Health, Nanjing Medical University, Jiangning District, Nanjing 211166, China; Jiangsu Province Engineering Research Center of Antibody Drug, Key Laboratory of Antibody Technique of National Health Commission, Nanjing Medical University, Nanjing 211166, China

**Keywords:** *ANO6* (TMEM16F), gastrointestinal stromal tumor, ferroptosis

## Abstract

Herein, we elucidate the potential role of *ANO6* (TMEM16F) in gastrointestinal stromal tumors (GISTs). *ANO6* expression in GIST and adjacent normal tissues was determined using reverse transcription-quantitative polymerase chain reaction (RT-qPCR) and western blotting. Cell proliferation, apoptosis, and pyroptosis were examined utilizing 3-(4,5-dimethylthiazol-2-yl)-2,5-diphenyltetrazolium bromide, terminal deoxynucleotidyl transferase dUTP Nick-End Labeling staining, and flow cytometry. In addition, the total iron and Fe^2+^ levels were assessed. IL-18 and IL-1β levels were also evaluated. Lipid reactive oxygen species (ROS), cystine (Cys), glutathione (GSH), and glutathione peroxidase 4 (GPX4) levels were evaluated using appropriate kits. Ferroptotic markers, including *Ptgs2*, *Chac1*, *SLC7A11*, and *SLC3A2*, were analyzed by RT-qPCR, western blotting, and immunohistochemistry. *ANO6* expression decreased in GIST tissues. *ANO6*-plasmid inhibits proliferation, induces apoptosis, and promotes pyroptosis in GIST-T1 and GIST-T1 IR cells. The *ANO6*-plasmid induced ferroptosis, as confirmed by enhanced lipid ROS levels, increased intracellular concentrations of total iron and Fe^2+^, promoted *Ptgs2* and *Chac1* expression, reduced Cys, GSH, and GPX4 levels, and downregulated *SLC7A11* and *SLC3A2* expression after *in vitro* and *in vivo* treatment with *ANO6*-plasmid. Moreover, the *ANO6*-plasmid inhibited GIST growth *in vivo*. Therefore, *ANO6* may be a promising therapeutic target for blocking the development of GIST via the induction of apoptosis, pyroptosis, and ferroptosis.

## 1 Introduction

Gastrointestinal stromal tumor (GIST) is a type of tumor originating from the mesenchymal tissue of the gastrointestinal tract, accounting for the majority of gastrointestinal mesenchymal tumors [1,2]. GIST, as a special type of tumor, is not sensitive to traditional chemotherapy and radiotherapy [3,4]. Surgical resection is considered the most effective treatment method [5]. Moreover, cancer immunotherapy is increasingly receiving attention due to breakthroughs in immune checkpoint inhibitors [6,7], and the immunotherapeutic strategies for GIST are growing fast [8]. Yeh et al. identified aurora kinase A as an unfavorable prognostic factor and a potential treatment target for metastatic GIST [9]. However, at present, the recurrence rate of GIST is high, and the survival rate of patients is poor. Therefore, it is necessary to develop new and more effective treatment strategies for patients with GIST.

Recently, there have been reports that several new types of programmed cell death, such as ferroptosis and pyroptosis, play important roles in regulating cancer progression and are considered a promising strategy for cancer treatment. Ferroptosis and cell pyroptosis are highly correlated with immune regulation in the tumor microenvironment. Ferroptosis is a newly discovered mode of cell death, and its development depends on intracellular free iron (Fe^2+^). Abnormal regulation of iron levels in cells can result in an imbalance between cell membrane redox and lipid peroxidation, eventually resulting in cell death [10]. Ferroptosis plays a vital role in the progression and development of several diseases [11,12]. In addition, antioxidants and glutathione peroxidase 4 (GPX4) are involved in the progression of ferroptosis. For instance, Zhou et al. found that ferroptosis is initiated by glutathione (GSH) depletion or *GPX4* inactivation [13]. The cystine (Cys)/glutamate antiporter system Xc^−^, which consists of *SLC7A11* and *SLC3A2*, is closely associated with ferroptosis [14]. Li et al. verified that inhibition of the *SLC7A11*-*GPX4* signaling pathway is involved in aconitine-induced ferroptosis *in vivo* and *in vitro* [15]. In recent years, ferroptosis has garnered enormous interest in cancer research communities. Ferroptosis can be seen in radiotherapy, chemotherapy, and tumor immunotherapy. Therefore, activation of ferroptosis may be a potential strategy to overcome the resistance mechanisms of traditional cancer treatments [10]. In addition, as a form of programmed cell death mediated by gasdermin, cell pyroptosis is a product of continuous cell expansion until cell membrane rupture, leading to the release of cell contents and activation of strong inflammatory and immune responses [16]. Pyroptosis is an innate immune response that can be triggered by various influencing factors that activate inflammasomes. Evidence has demonstrated that pyroptosis exerts benefits on cancer immunotherapies [17]. However, the specific mechanisms underlying ferroptosis and pyroptosis in GIST require further investigations.

*ANO6* (TMEM16F) is a protein with ten transmembrane segments that exists in various tissues and cells [18]. Studies have shown that *ANO6* (TMEM16F) plays important roles in cell growth and migration [19]. Zhao et al. found that *ANO6* (TMEM16F) inhibition limited pain-associated behavior and improved motor function by promoting microglial M2 polarization in mice [20]. Bricogne et al. found that *ANO6* (TMEM16F) activation by Ca(^2+^) triggers plasma membrane expansion and directs PD-1 trafficking [21]. Lin et al. found that TMEM16F/*ANO6* is negatively regulated by the actin cytoskeleton and intracellular MgATP [22]. Moreover, evidence indicates that activation of *ANO6* (TMEM16F) contributes to various forms of regulated cell death [23]. However, the expression and role of *ANO6* (TMEM16F) in GISTs remains unclear.

Thus, our study was designed to illustrate the functions of *ANO6* (TMEM16F) in GIST ferroptosis and elucidate its potential mechanism. Our findings provide the first evidence that ANO6 (TMEM16F) inhibits GIST growth and induces ferroptosis by regulating *SLC7A11* and *SLC3A2C* expression, thereby providing a therapeutic basis for GIST treatment.

## 2 Methods

### 2.1 Clinical specimen collection

GIST and adjacent normal tissues were collected from 15 patients with GIST at the The Yangzhou School of Clinical Medicine of Nanjing Medical University. All specimens were rapidly frozen and stored in liquid nitrogen and preserved at −80°C for further analysis.

### 2.2 Cell culture

GIST-T1 cells were bought from the American Type Culture Collection and cultured in Dulbecco’s modified Eagle’s medium (Thermo Fisher) supplemented with 10% fetal bovine serum (HyClone, Logan, UT, USA) and 100 U/mL penicillin and 100 μg/mL streptomycin at 37°C in a humid atmosphere containing 5% CO_2_.

### 2.3 Construction of drug-resistant cell lines

Intermittent imatinib (IM) administration was performed on cells in the logarithmic growth phase. After 48 h of treatment, a drug-free culture medium was used instead of the culture medium. Specific IM concentrations were administered until normal cell growth was observed. Subsequently, the concentration was increased, and the above process was repeated to obtain drug-resistant cell lines.

### 2.4 Cell transfection

GIST-T1 and GIST-T1 IR cells were induced by 1 µg control-plasmid (sc-437275; Santa Cruz Biotechnology) and 1 µg *ANO6*-plasmid (sc-402736-ACT Santa Cruz Biotechnology) using Lipofectamine 2000 (11668019; Thermo Fisher) for 48 h following the manufacturer’s protocol. The cells were harvested after transfection.

### 2.5 3-(4,5-Dimethylthiazol-2-yl)-2,5-diphenyltetrazolium bromide (MTT) assay

After treatment, GIST-T1 and GIST-T1 IR cells were implanted into 96-well plates and cultured for 24 h at 37°C. Then, cells were treated with 10 μL of MTT solution and continuously incubated for a further 4 h. Afterward, the solution was removed and 100 μL of dimethyl sulfoxide was added to each well to solubilize the formazan product. Finally, the optical density at 570 nm was measured using a microplate reader (BioTek, Richmond, USA) after 15 min of vibration mixing, following the manufacturer’s instructions.

### 2.6 Flow-cytometry analysis

After treatment, GIST-T1 and GIST-T1 IR cells were implanted into 96-well plates and cultured for 24 h at 37°C. The cells were collected by centrifugation at 4°C for 5 min. The cells were then washed with phosphate-buffered saline (PBS). Apoptosis was detected using the Annexin V-FITC/PI Apoptosis Detection Kit (Beyotime, Beijing, China) following the manufacturer’s instructions. Apoptosis was assessed using a BD Aria III flow cytometer (BD Technologies). Pyroptosis in GIST-T1 and GIST-T1 IR cells was determined by flow cytometry according to the manufacturer’s instructions.

### 2.7 Reactive oxygen species (ROS) assay

2'-7'-Dichlorodihydrofluorescein diacetate (DCFH-DA) was used to quantify the ROS levels according to the protocol of the ROS fluorescence assay kit (E-BC-K138-F; Elabscience). GIST-T1 and GIST-T1 IR cells were plated in six-well plates and incubated for 24 h. After washing with PBS for three times, cells were labeled with 5 μM DCFH-DA under standard conditions for 30 min, cells were collected, and fluorescence intensity of DCF was detected using a microplate reader (SMR16.1, USCNK).

### 2.8 Iron assay

The Iron Assay Kit (E-BC-K772-M/E-BC-K773-M; Elabscience) was used to detect the total iron or Fe^2+^ levels in GIST-T1 and GIST-T1 IR cells. According to the manufacturer’s protocol, the iron assay buffer and iron reducer were sequentially added to the cells. The samples were thoroughly mixed in the dark, added to an iron-reducing agent and iron probe, and cultured for 30 min. Intracellular ferrous levels were quantified using a kit, and the absorbance at 593 nm was calculated as the intracellular Fe^2+^ level.

### 2.9 Cys, GSH level, and GPX4 activity measurements

GIST-T1 and GIST-T1 IR cells were collected after treatment with dimethyl sulfoxide or a specified concentration of the drug for 24 h, according to the manufacturer’s instructions. Cys and GSH levels were detected using a human Cys enzyme-linked immunosorbent assay (ELISA) kit (ELK9092; ELK Biotechnology) and a GSH Assay Kit (A006-2; Nanjing Jiancheng Biotechnology). The GPX4 activity was measured using a Human GPX4 ELISA Kit (ELK4775; ELK Biotechnology).

### 2.10 ELISA

After GIST-T1 and GIST-T1 IR cells were transfected with the control plasmid and *ANO6*-plasmid, we centrifuged and collected the supernatant of the cells for ELISA. Then, the levels of IL-18 (ELK1245; ELK Biotechnology) and IL-1β (ELK1270; ELK Biotechnology) were assessed following the manufacturer’s instructions.

### 2.11 Reverse transcription-quantitative polymerase chain reaction (RT-qPCR) assay

Total RNA was extracted from GIST cells and GIST-T1 IR lines or tissues using the TRIzol Total RNA Extraction Reagent (EP013; ELK Biotechnology) according to the manufacturer’s protocol. The levels of *ANO6*, *Bax*, *Bcl-2*, *SLC7A11*, *SLC3A2*, *Ptgs2*, and *Chac1* were measured using RT-qPCR. Total RNA was reverse transcribed into cDNA following the instructions of EntiLink 1st Strand cDNA Synthesis Super Mix (EQ031; ELK Biotechnology), and RT-qPCR analysis was conducted using EnTurbo SYBR Green PCR SuperMix (EQ001; ELK Biotechnology) with an ABI 7500 Real-Time PCR System (Applied Biosystems). Target gene expressions were calculated using the 2^−ΔΔCt^ method [24]. Primer sequences for PCR are listed in Table 1.

**Table 1:** Primer sequences for PCR

Gene	Sequences (5′–3′)	
GAPDH	sense	CATCATCCCTGCCTCTACTGG
	antisense	GTGGGTGTCGCTGTTGAAGTC
Ptgs2	sense	AGATTATGTGCAACACTTGAGTGG
	antisense	ATTCCTACCACCAGCAACCCT
Chac1	sense	GTGGTGACGCTCCTTGAAGAT
	antisense	GCCTCTCGCACATTCAGGTAC
SLC7A11	sense	TGTGGGGTCCTGTCACTATTTG
	antisense	GATATCACAGCAGTAGCTGCAGG
Bax	sense	TCTGAGCAGATCATGAAGACAGG
	antisense	ATCCTCTGCAGCTCCATGTTAC
Bcl-2	sense	AGGATTGTGGCCTTCTTTGAG
	antisense	AGCCAGGAGAAATCAAACAGAG
SLC3A2	sense	CGATTACCTGAGCTCTCTGAAG
	antisense	TAAGGTCCAGAATGACACGGAT

### 2.12 Western blot assay

GIST-T1 and GIST-T1 IR cell proteins were extracted using radioimmunoprecipitation assay buffer (AS1004; ASPEN) and evaluated using a bicinchoninic acid assay kit (AS1086; ASPEN). Average protein concentrations were separated by 10% sodium dodecyl sulfate-polyacrylamide gel electrophoresis and transferred to a polyvinylidene fluoride membrane. Then, the membrane was blocked in 5% skim milk for 2 h at room temperature and incubated in specific antibodies against glyceraldehyde-3-phosphate dehydrogenase (GAPDH) (ab181602, 1:10,000 dilution; Abcam), *ANO6* (20784-1-AP, 1:1,000 dilution; Wuhan Sanying Biotechnology), *Bax* (50599-2-Ig, 1:2,000 dilution; Wuhan Sanying Biotechnology), *Bcl-2* (ab321124, 1:1,000 dilution; Abcam), *SLC7A11* (26864-4-AP, 1:1,000 dilution; Wuhan Sanying Biotechnology), *SLC3A2* (15193-1-AP, 1:5,000 dilution; Wuhan Sanying Biotechnology), GSDMD-N (A22523, 1:1,000 dilution; abclonal), or cleaved-Caspase1 (AF4005, 1:500 dilution; affbiotech) overnight at 4°C. Membranes were then incubated with secondary antibodies (AS1107, 1:10,000 dilution; ASPEN) for 1 h. Finally, signals were developed using electrochemiluminescence detection system reagents (AS1059; ASPEN) according to the manufacturer’s instructions.

### 2.13 Animal studies

All mice were placed in a specific pathogen-free environment with a standard light–dark cycle of 25°C for 12 h and had free access to food and water. Then, GIST-T1 cells were injected subcutaneously into nude mice with 100 μL of normal saline. The tumor volume (*V*) was calculated using the formula: *V* = *L* × *W* ^2^/2, where *L* is the tumor length and *W* is the tumor width. Tumors were collected and weighed from all mice after euthanasia. This study is reported in accordance with ARRIVE guidelines. Animal care and experimental procedures were approved by the Animal Ethics Committee of Yangzhou University (Approval number: 202111020).

### 2.14 Immunohistochemical analysis

Tumor samples were fixed with 4% paraformaldehyde at room temperature for 24 h, dehydrated, and waxed. Then, the sample was cut into 2–3 μm slices. The slices were placed in 0.01 M citrate buffer (pH 6.0), heated in a microwave oven for antigen repair on medium heat for 2–8 min. The slices were exposed to 3% H_2_O_2_ for 15–30 min to bleach the endogenous peroxidase and then rinsed in PBS for three times. Then, a sufficient amount of diluted primary antibodies against *ANO6*, *SLC7A11*, or *SLC3A2* were added to each slice and incubated overnight at 4°C. These sections were then incubated with the secondary antibody for 30 min. The slices were examined using light microscopy (Olympus).

### 2.15 Terminal deoxynucleotidyl transferase dUTP nick-end labeling (TUNEL) analysis

The tissue of GIST was fixed with 4% paraformaldehyde for more than 24 h, and 2–3 μm paraffin sections were taken after dehydration. The slices were then dewaxed in xylene for 5–10 min, washed with anhydrous ethanol for 5 min, soaked in 0.2% Triton X-100 for 15 min, and soaked in 3,3′-diaminobenzidine solution for 30 min. Images were analyzed using Image-Pro Plus 6.0 software (Media Cybernetics, Inc.), and the slices were photographed and counted under an optical microscope.

### 2.16 Statistical analysis

Statistical analyses were conducted using SPSS 19.0 (SPSS Inc., Chicago, IL, USA). We used the Kolmogorov–Smirnov test to determine the normality of the data in SPSS. All findings are displayed by mean ± standard deviation from three independent experiments. Mean differences among groups were estimated using the unpaired Student’s *t*-test or one-way analysis of variance with Tukey’s *post hoc* test. Statistical significance was set at *P* < 0.05.


**Ethics approval and consent to participate:** This study is reported in accordance with ARRIVE guidelines. The research procedure was approved by the ethics committee of The Yangzhou School of Clinical Medicine of Nanjing Medical University (Approval number: 2022ky152) in accordance with the Declaration of Helsinki. Animal care and experiment procedures are approved by the Animal Ethics Committee of Yangzhou University (Approval number. 202111020).
**Informed consent:** All patients signed an informed consent form and approved the use of their tissues in the study.

## 3 Results

### 3.1 *ANO6* (TMEM16F) (ANO6) was low-expressed in the stromal tumor tissues of patients with GISTs

**Figure 1:**
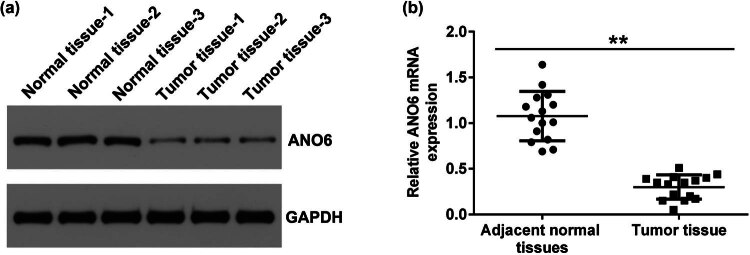
Expression of *ANO6* (TMEM16F) in stromal tumor tissues and adjacent normal tissues of patients with GIST. Relative levels of *ANO6* (TMEM16F) in stromal tumor tissues and adjacent normal tissues from patients with GISTs were evaluated by (a) western blot assay and (b) RT-qPCR. **P < 0.01.

Stromal tumors and adjacent normal tissues were obtained from 15 patients with GISTs. The levels of *ANO6* (TMEM16F) were analyzed using RT-qPCR and western blotting. As presented in Figure 1a and b, the levels of *ANO6* (TMEM16F) were remarkably lower in the stromal tumor tissues of patients with GIST than in the adjacent normal tissues, indicating a regulatory role of *ANO6* (TMEM16F) in GIST.

### 3.2 *ANO6* (TMEM16F) was downregulated in the IM‑resistant GIST‑T1 IR cell line

**Figure 2:**
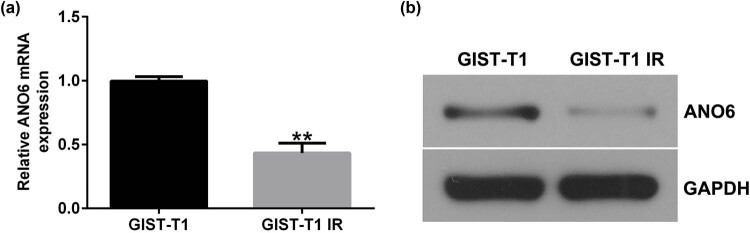
Expression of *ANO6* (TMEM16F) in GIST-T1 and GIST-T1 IR cells. The relative levels of *ANO6* (TMEM16F) in GIST-T1 and GIST-T1 IR cells were evaluated by (a) RT-qPCR and (b) western blotting, respectively. **P < 0.01.

Moreover, we detected *ANO6* (TMEM16F) expression in GIST-T1 IR and GIST-T1 cells. The RT-qPCR and western blotting results suggested that the level of *ANO6* (TMEM16F) was lower in GIST-T1 IR cells than in GIST-T1 cells (Figure 2a and b). Our data indicate that *ANO6* (TMEM16F) is involved in the regulation of IM-resistant GIST-T1 IR cell line.

### 3.3 Upregulation of *ANO6* (TMEM16F) inhibited GIST-T1 cell proliferation and induced cell apoptosis

**Figure 3:**
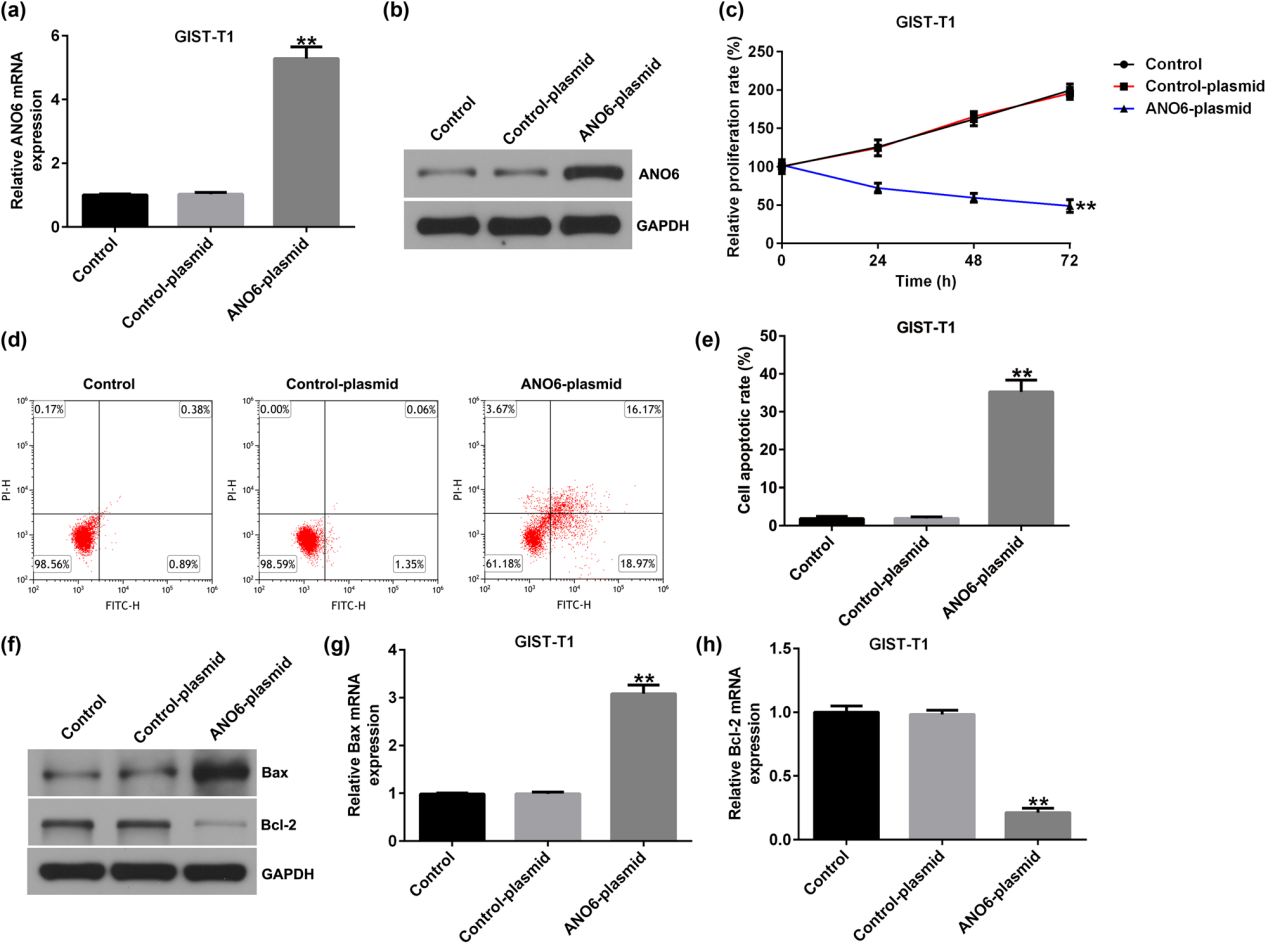
Effects of *ANO6* (TMEM16F) on GIST-T1 cell proliferation and apoptosis. GIST-T1 cells were transfected with the control-plasmid and the *ANO6*-plasmid. (a) and (b) RT-qPCR and western blot analysis of *ANO6* (TMEM16F) levels in the control-plasmid and *ANO6*-plasmid groups. (c) The growth of GIST-T1 cells was assessed by MTT. (d) Apoptosis was determined by flow cytometry. (e) Quantification of apoptotic GIST-T1 cells. (f) Western blotting analysis of *Bax* and *Bcl-2* expression. (g) and (h) *Bax* and *Bcl-2* mRNA levels were determined using RT-qPCR. **P < 0.01.

To further illustrate the mechanism of *ANO6* (TMEM16F) (ANO6) in GIST, we transfected GIST-T1 cells with a control-plasmid and *ANO6*-plasmid. Results from RT-qPCR and western blot assays suggested that *ANO6* (TMEM16F) was upregulated in *ANO6*-plasmid transfected GIST-T1 cells compared to the control plasmid group (Figure 3a and b). In addition, the results of MTT and flow cytometry assays revealed that the upregulation of *ANO6* (TMEM16F) inhibited the proliferation of GIST-T1 cells (Figure 3c) and increased the number of apoptotic GIST-T1 cells (Figure 3d and e). We also determined the levels of apoptosis-related proteins, including *Bax* and *Bcl-2*, by RT-qPCR and western blotting. The *ANO6*-plasmid increased *Bax* expression (Figure 3f and g) and reduced *Bcl-2* expression (Figure 3f and h). Our data revealed that *ANO6* (TMEM16F) was involved in GIST progression by regulating GIST-T1 cell proliferation and apoptosis.

### 3.4 Overexpression of *ANO6* (TMEM16F) suppressed GIST-T1 IR cell proliferation and promoted cell apoptosis

**Figure 4:**
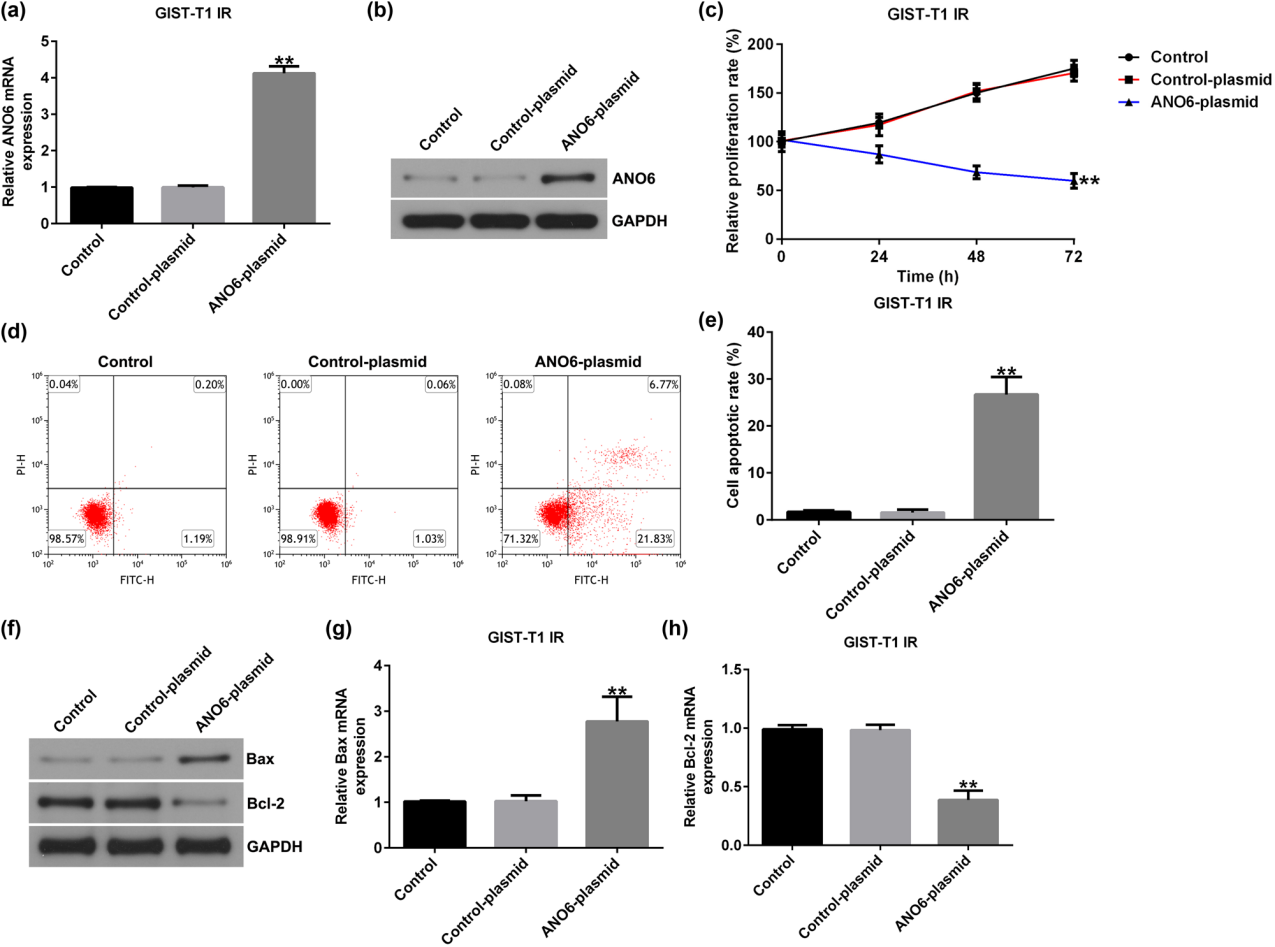
Effects of *ANO6* (TMEM16F) on GIST-T1 IR cell proliferation and apoptosis. Control-plasmid and *ANO6*-plasmid were transfected into GIST-T1 IR cells. (a) and (b) *ANO6* (TMEM16F) levels were determined by RT-qPCR and western blot analysis. (c) GIST-T1 IR cell viability was assessed by MTT. (d) Apoptosis was determined by flow cytometry. (e) Quantification of apoptotic GIST-T1 IR cells. (f) Western blotting analysis of *Bax* and *Bcl-2* expression. (g) and (h) *Bax* and *Bcl-2* mRNA levels were determined using RT-qPCR. **P < 0.01.

We also investigated the roles of the *ANO6*-plasmid in GIST-T1 IR cell proliferation and apoptosis. We found that the *ANO6*-plasmid upregulated *ANO6* (TMEM16F) expression in GIST-T1 IR cells, compared to the control-plasmid group (Figure 4a and b). Moreover, the *ANO6*-plasmid led to suppressed GIST-T1 IR cell proliferation (Figure 4c) and enhanced apoptosis (Figure 4d and e). Furthermore, RT-qPCR and western blotting assays revealed that the *ANO6*-plasmid increased *Bax* expression (Figure 4f and g) and reduced *Bcl-2* expression (Figure 4f and h) in GIST-T1 IR cells, indicating that *ANO6* (TMEM16F) is a vital regulator of GIST progression.

### 3.5 *ANO6*-plasmid promotes pyroptosis in GIST-T1 and GIST-T1 IR cells

**Figure 5:**
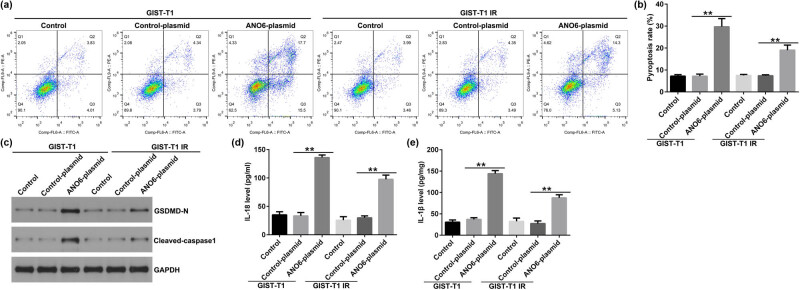
Effects of *ANO6* (TMEM16F) on GIST-T1 and GIST-T1 IR cell pyroptosis. (a) and (b) Pyroptosis of GIST-T1 and GIST-T1 IR cells was confirmed by flow cytometry. (c) Western blot analysis of GSDMD-N and cleaved-caspase 1 expression. (d) and (e) The levels of IL-18 and IL-1β were assessed using ELISA assay. **P < 0.01.

Mechanistically, we explored the effects of *ANO6* on GIST-T1 and GIST-T1 IR cell pyroptosis. As shown in Figure 5a and b, the *ANO6*-plasmid induced GIST-T1 and GIST-T1 IR cells. Furthermore, the effector molecules of pyroptosis, including GSDMD-N and cleaved caspase 1, were determined by western blotting. We observed that GSDMD-N and cleaved caspase 1 density in the *ANO6*-plasmid group was remarkably increased relative to the control-plasmid group (Figure 5c). ELISA for *IL-18* and *IL-1β* expression levels were also evaluated. Results from Figure 5d and e revealed that the *ANO6*-plasmid increased *IL-18* and *IL-1β* expressions, as opposed to the control-plasmid. These results suggested that the *ANO6*-plasmid increased pyroptosis-related markers, indicating a promotional effect on pyroptosis.

### 3.6 *ANO6* (TMEM16F) induced ferroptosis by regulating *SLC7A11* and *SLC3A2* in GIST-T1 cells

**Figure 6:**
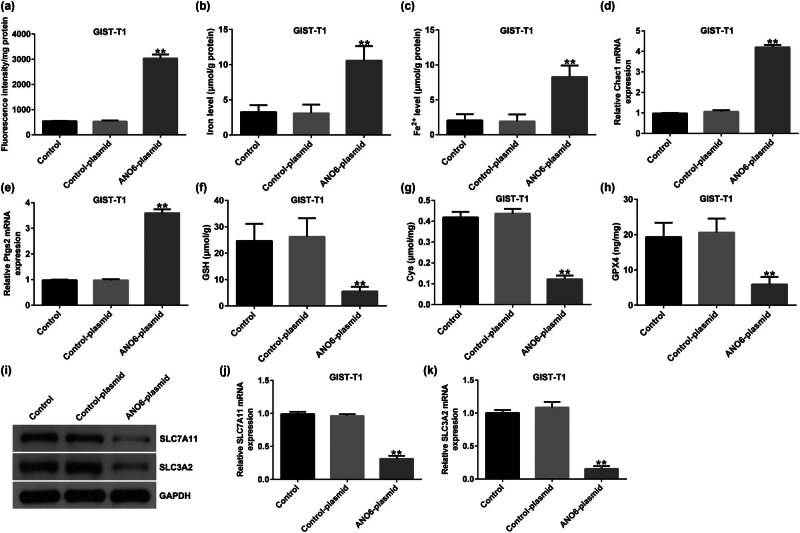
Effects of *ANO6* (TMEM16F) on GIST-T1 cell ferroptosis. GIST-T1 cells were transfected with the control-plasmid and the *ANO6*-plasmid. (a) Generation of lipid ROS in GIST-T1 cells was detected by flow cytometry. Total iron (b) and ferrous iron (c) levels were evaluated in GIST-T1 cells after treatment with the control-plasmid or the *ANO6*-plasmid. (d) and (e) Levels of *Ptgs2* and *Chac1* were detected by RT-qPCR. Determination of Cys (f), GSH (g), and GPX4 (h) levels in GIST-T1 cells. (i) Detection of *SLC7A11* and *SLC3A2* expression in the control, control-plasmid, and *ANO6*-plasmid groups. (j) and (k) mRNA levels of *SLC7A11* and *SLC3A2* were assessed using RT-qPCR. **P < 0.01 vs control-plasmid.

Ferroptosis is an iron-dependent process that is different from necrosis and apoptosis. An increasing number of studies have suggested that ferroptosis is a critical modality in cancer-related deaths [25]. We explored the effects of the *ANO6*-plasmid on ferroptosis in GIST-T1 cells. We found that the upregulation of *ANO6* (TMEM16F) enhanced lipid ROS levels (Figure 6a) and increased the intracellular concentrations of total iron (Figure 6b) and Fe^2+^ (Figure 6c) in GIST-T1 cells. In addition, we determined the markers of ferroptosis, including *Ptgs2* and *Chac1*. Our data revealed that *Ptgs2* and *Chac1* were upregulated in *ANO6*-plasmid transfected GIST-T1 cells (Figure 6d and e). Further mechanistic experiments indicated that the *ANO6*-plasmid reduced Cys, GSH, and GPX4 levels as opposed to the control-plasmid group (Figure 6f–h), indicating that *ANO6* (TMEM16F) inhibits GIST growth and induces ferroptosis in GIST-T1 cells.

Previous reports have revealed that ferroptosis is linked to multiple diseases and results from iron-dependent lipid peroxidation after inactivation of *SLC7A11* and *SLC3A2* [26]. Next, we evaluated whether *ANO6* (TMEM16F) affected *SLC7A11* and *SLC3A2* expression in GIST-T1 cells. As shown in Figure 6I-K, *SLC7A11* and *SLC3A2* were downregulated in *ANO6*-plasmid transfected cells. Our data indicate that *ANO6* (TMEM16F)-induced ferroptosis is mediated by *SLC7A11* and *SLC3A2*.

### 3.7 *ANO6* (TMEM16F) stimulated ferroptosis by regulating *SLC7A11* and *SLC3A2* in GIST-T1 IR cells

**Figure 7:**
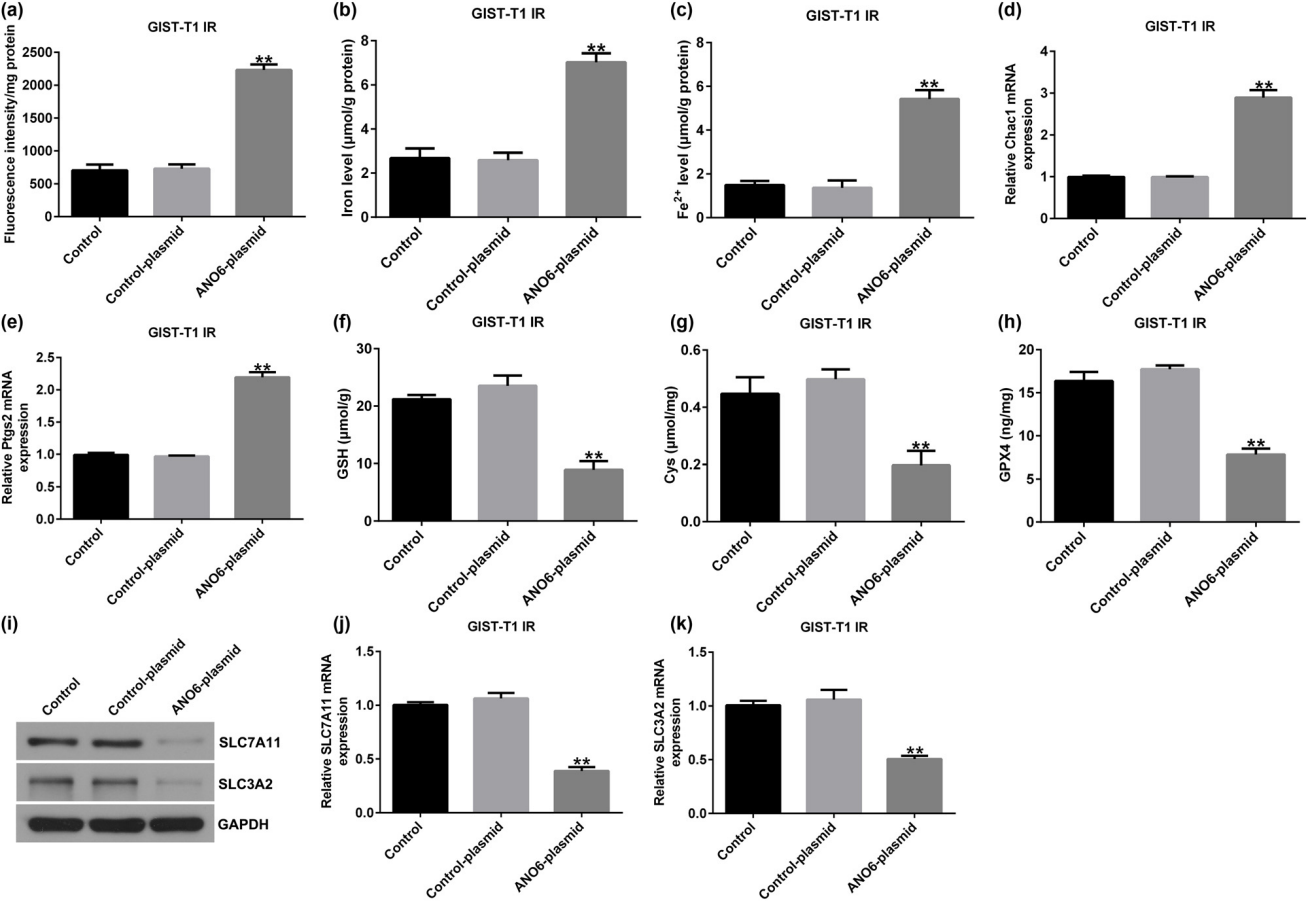
Effects of *ANO6* (TMEM16F) on GIST-T1 IR cell ferroptosis. GIST-T1 IR cells were transfected with the control-plasmid and the *ANO6*-plasmid. (a) The generation of lipid ROS was detected using flow cytometry. Total iron (b) and ferrous iron (c) were evaluated in GIST-T1 IR cells after treatment with the control-plasmid or the *ANO6*-plasmid. (d) and (e) Levels of *Ptgs2* and *Chac1* were detected by RT-qPCR. Determination of Cys (f), GSH (g), and GPX4) (h) levels in GIST-T1 IR cells. (i) Detection of *SLC7A11* and *SLC3A2* expression in the control, control-plasmid, and *ANO6*-plasmid groups. (j) and (k) mRNA levels of *SLC7A11* and *SLC3A2* were assessed using RT-qPCR. **P < 0.01 vs control-plasmid.

Furthermore, we investigated the roles of the *ANO6*-plasmid in the ferroptosis of GIST-T1 IR cells. Our data revealed that the *ANO6*-plasmid increased lipid ROS (Figure 7a), iron (Figure 7b), and Fe^2+^ levels (Figure 7c) in GIST-T1 IR cells. Furthermore, qRT-PCR analysis suggested that *Ptgs2* and *Chac1* expression was enhanced after *ANO6*-plasmid transfection (Figure 7d and e). Moreover, we detected ferroptosis-related gene expression in *ANO6*-plasmid transfected GIST-T1 IR cells, and our data demonstrated that the upregulation of *ANO6* inhibited Cys, GSH, and GPX4 levels (Figure 7f–h), as well as *SLC7A11* and *SLC3A2* expression (Figure 7i–k), compared to the control-plasmid group. Our data revealed that *ANO6* (TMEM16F) suppresses GIST growth and induces ferroptosis by regulating *SLC7A11* and *SLC3A2*.

### 3.8 *ANO6*-plasmid inhibited GIST growth *in vivo*

**Figure 8:**
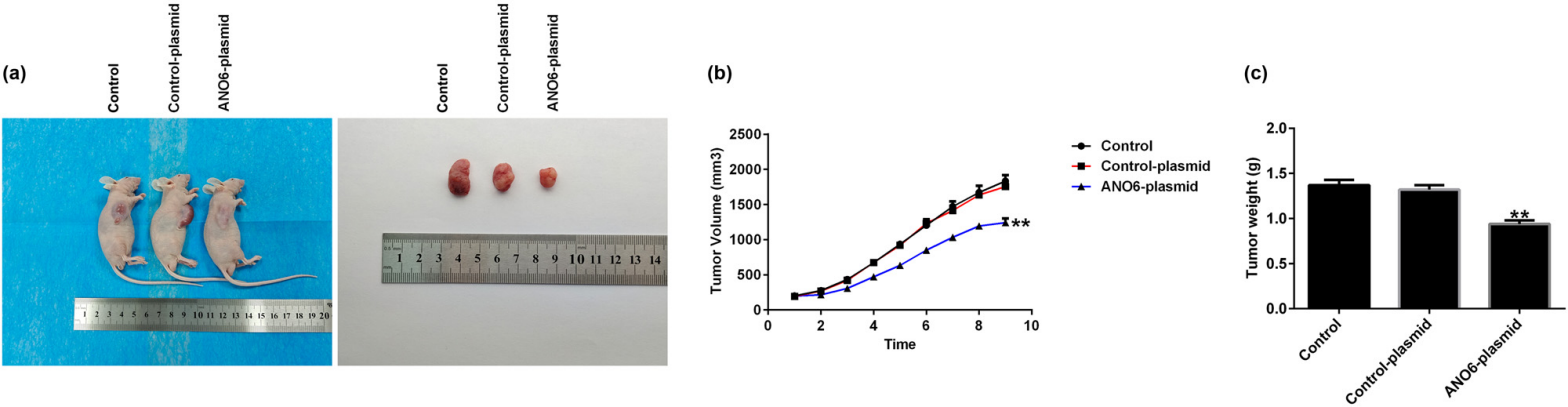
Effects of *ANO6* (TMEM16F) on the growth of xenograft *in vivo*. (a) Gross appearance of tumor. (b) and (c) Average tumor volume and body weight changes in each group were calculated. **P < 0.01 vs control-plasmid.

We performed *in vivo* experiments to analyze the regulatory role of *ANO6* (TMEM16F) in GIST progression. The xenograft tumor models were treated with the *ANO6*-plasmid, and Figure 8a shows a representative diagram of the xenografts. Furthermore, we calculated tumor volumes and weights of the xenografts. We found that tumor volumes and weights were reduced in nude mice inoculated with GIST-T1 cells after *ANO6*-plasmid treatment (Figure 8b and c). In summary, our data suggested that the *ANO6*-plasmid blocked GIST growth.

### 3.9 *ANO6* (TMEM16F) regulated *SLC7A11* and *SLC3A2* expression in GIST *in vivo*

**Figure 9:**
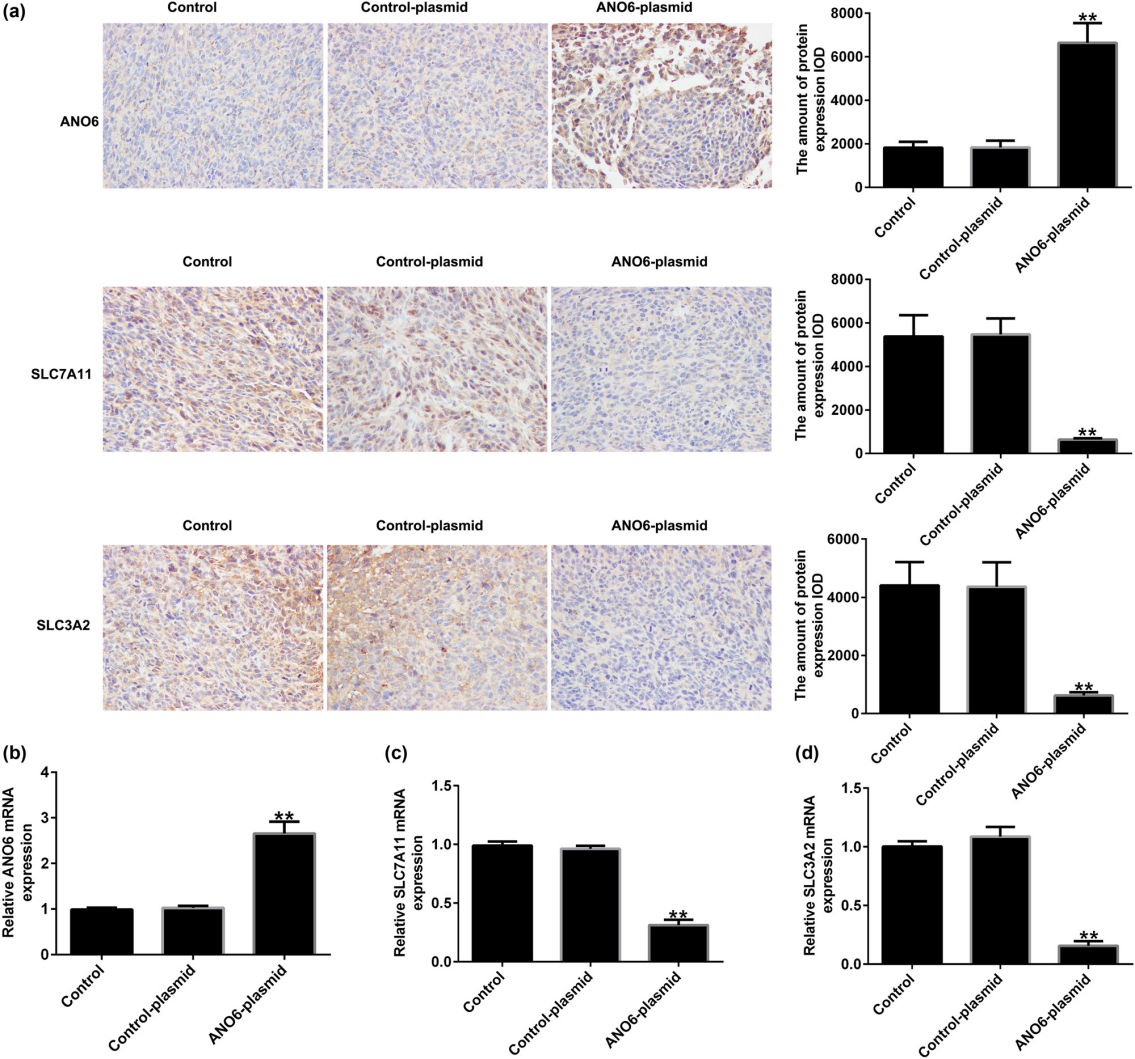
Effects of *ANO6* (TMEM16F) on *SLC7A11* and *SLC3A2* expression in GIST *in vivo.* (a) The expression of *ANO6* (TMEM16F), *SLC7A11*, and *SLC3A2* in GIST tissues was determined using IHC assays. (b)–(d) mRNA expression of *ANO6* (TMEM16F), *SLC7A11*, and *SLC3A2* in GISTs tissues was determined by RT-qPCR. **P < 0.01 vs control-plasmid.

We assessed the effect of *ANO6* (TMEM16F) on *SLC7A11* and *SLC3A2* expression in GIST *in vivo*. Immunohistochemistry suggested that the *ANO6*-plasmid prominently increased *ANO6* (TMEM16F) expression (Figure 9a) and obviously reduced *SLC7A11* and *SLC3A2* expression (Figure 9a) compared to the control-plasmid group. Similar results were obtained using RT-qPCR (Figure 9b–d). These findings demonstrate that *ANO6* (TMEM16F) inhibits GIST growth by regulating *SLC7A11* and *SLC3A2* expression.

### 3.10 *ANO6* (TMEM16F) induced cell apoptosis in GIST *in vivo*

**Figure 10:**
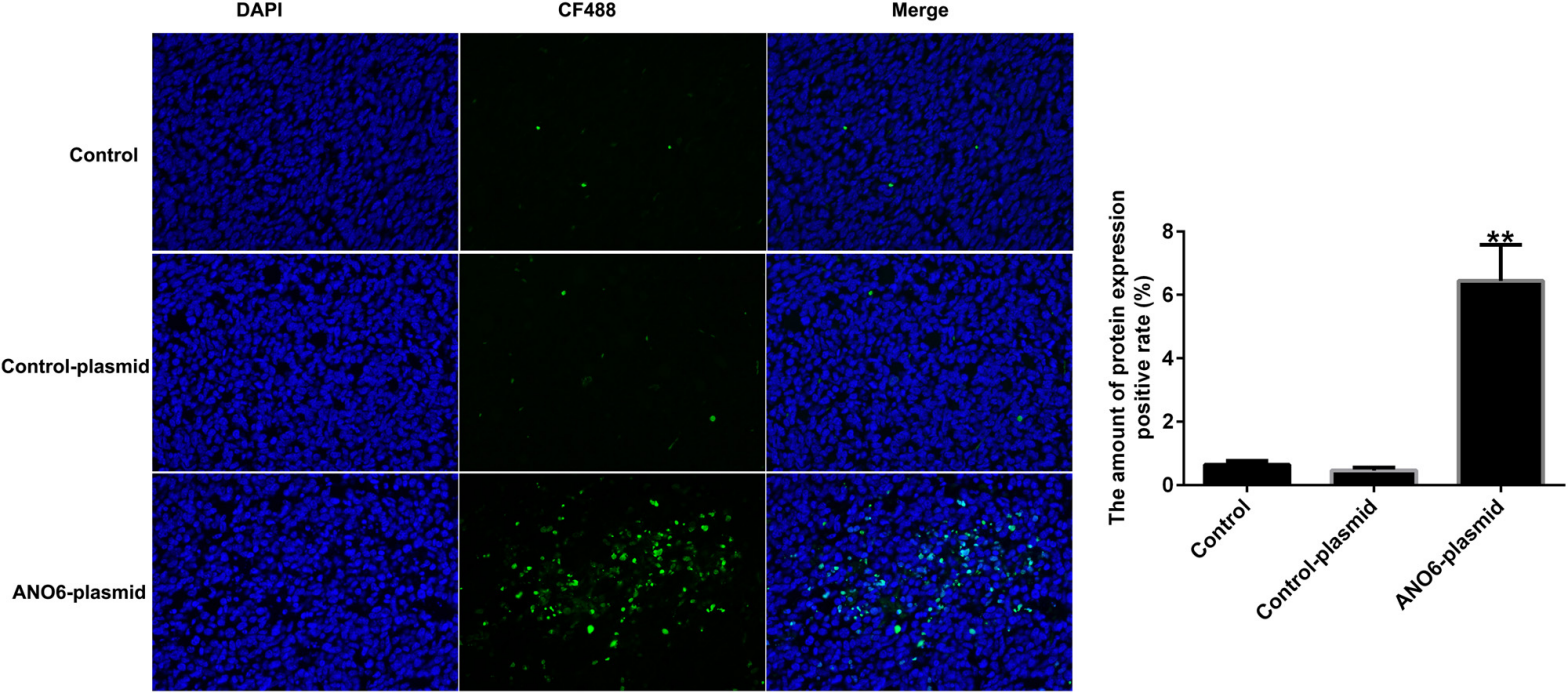
Effects of *ANO6* (TMEM16F) on GIST cell apoptosis *in vivo.* TUNEL images of tumor samples and quantitative analysis of apoptosis rates. **P < 0.01 vs control-plasmid.

To further illustrate the mechanism by which *ANO6* (TMEM16F) inhibited tumor growth *in vivo*, we determined the number of apoptotic cells in GIST. As shown in Figure 10, TUNEL staining suggested that the *ANO6*-plasmid significantly increased TUNEL-positive GIST-T1 cells compared to control-plasmid transfected cells, suggesting that *ANO6* (TMEM16F) plays a pro-apoptotic role in GIST.

### 3.11 *ANO6* (TMEM16F) inhibited GIST growth by inducing ferroptosis *in vivo*

**Figure 11:**
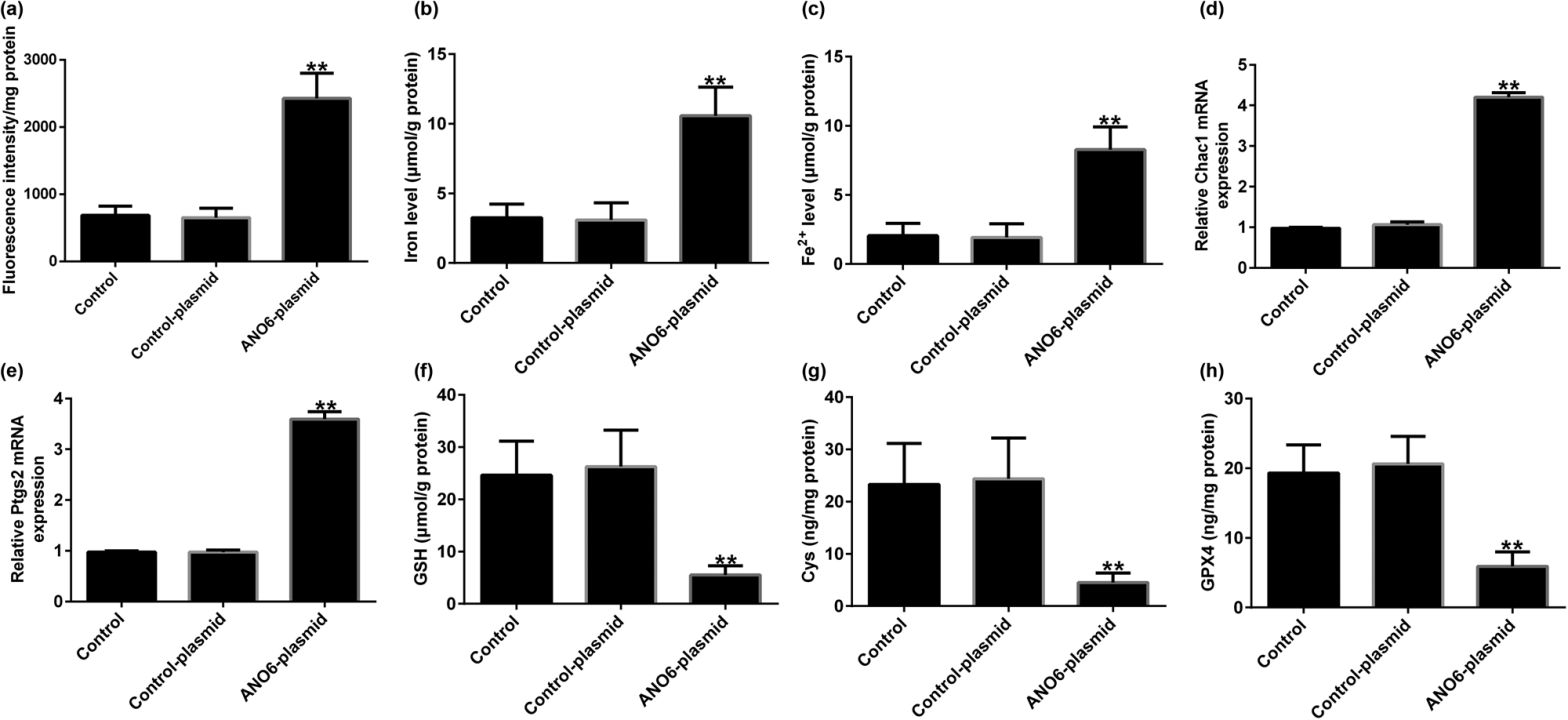
Effects of *ANO6* (TMEM16F) on GIST cell ferroptosis *in vivo*. (a) Lipid ROS stimulation was detected using an ROS fluorescence assay kit. Total iron (b) and ferrous iron (c) were analyzed after treatment with the control-plasmid or the *ANO6*-plasmid. (d) and (e) RT-qPCR analysis of *Ptgs2* and *Chac1* mRNA levels. Detection of Cys (f), GSH (g), and GPX4 (h) in GISTs. **P < 0.01 vs control-plasmid.

Finally, we evaluated the effect of *ANO6* (TMEM16F) on the ferroptosis of GIST *in vivo*. Our data demonstrated that the *ANO6*-plasmid remarkably enhanced lipid ROS levels (Figure 11a), intracellular concentrations of total iron (Figure 11b), and Fe^2+^ levels (Figure 11c). RT-qPCR analysis revealed that the *ANO6*-plasmid enhanced *Ptgs2* and *Chac1* expression levels as opposed to the control-plasmid group (Figure 11d and e). In addition, reduced Cys, GSH, and GPX4 expression levels were observed in *ANO6*-plasmid treated xenograft tumor models (Figure 11f–h). Our findings revealed that *ANO6* (TMEM16F) inhibited GIST growth and induced ferroptosis by regulating *SLC7A11* and *SLC3A2* expression.

## 4 Discussion

This study indicated that *ANO6* (TMEM16F) is abnormally low expressed in GIST, which can inhibit the growth of GIST *in vitro* and *in vivo*, inducing cell pyroptosis and ferroptosis. It is a potential therapeutic target for GIST.

GISTs are a type of mesenchymal tumors that originate from the precursors of gastrointestinal connective histiocytes and often occur in middle-aged and elderly individuals [27]. IM has been used as a first-line treatment for patients with GIST with metastatic recurrence or unresectability [28,29]. Although many patients benefit from IM, some exhibit drug resistance within 18–24 months of treatment, leading to disease progression and even death [30]. However, GISTs have a high recurrence rate and poor survival rate, and there is currently no effective method for treating advanced metastatic diseases. Drug resistance remains unclear. Therefore, there is an urgent need to identify novel therapeutic targets against GIST. According to reports, TMEM16A/*ANO1* and TMEM16F/*ANO6* are regulated by intracellular Ca^2+^ and plasma membrane phospholipids, while TMEM16F/*ANO6* is a phospholipid scramjet enzyme that also generates Cl^−^ current [31]. In addition, activation of *ANO6* (TMEM16F) contributes to various forms of regulatory cell death in diseases. Cui et al. suggested that *ANO6* (TMEM16F) may be a new therapeutic target for Alzheimer’s disease [32]. Research has confirmed the important regulatory role of *ANO6* (TMEM16F) in cell growth and migration [19]. However, the expression and role of *ANO6* (TMEM16F) in GIST and GIST-T1 IR cells remain unclear.

First, we determined the *ANO6* (TMEM16F) expression levels in stromal tumor tissues and adjacent normal tissues. Our data revealed that *ANO6* (TMEM16F) was expressed at low levels in stromal tumor tissues from patients with GISTs compared to those in adjacent normal tissues, indicating that *ANO6* (TMEM16F) is related to the progression of GIST. Further mechanistic experiments suggested that the *ANO6*-plasmid upregulated *ANO6* (TMEM16F) levels compared with the control-plasmid group. Moreover, the *ANO6*-plasmid inhibited the proliferation of GIST-T1 and GIST-T1 IR cells and increased the number of apoptotic cells. Zhao et al. reported that ligustrazine suppresses neuronal apoptosis via the *Bax*/*Bcl-2* and caspase-3 pathways in PC-12 cells and rats with vascular dementia [33]. Wei et al. suggested that borax-induced apoptosis in HepG2 cells involves *p53*, *Bcl-2*, and *Bax* [34]. Therefore, we determined the effects of the *ANO6*-plasmid on *Bax* and *Bcl-2*. Our data revealed that the *ANO6*-plasmid increased *Bax* expression and reduced *Bcl-2* expression, indicating that *ANO6* (TMEM16F) is involved in GIST progression by regulating GIST-T1 and GIST-T1 IR cell proliferation and apoptosis. Pyroptosis is another form of programmed inflammatory cell death associated with various pathologies [35–37]. We further determined the effects of the *ANO6*-plasmid on GIST-T1 and GIST-T1 IR cells. Our data revealed that the *ANO6*-plasmid enhances pyroptosis, as confirmed by increased GSDMD-N, cleaved-caspase 1, IL-18, and IL-1β expressions.

Further *in vivo* experiments confirmed the regulatory role of *ANO6* (TMEM16F) in GIST progression, as evidenced by the reduction in tumor volume and weights after *ANO6*-plasmid treatment. Moreover, TUNEL staining suggested that the *ANO6*-plasmid significantly increased TUNEL-positive GIST-T1 cells compared to control-plasmid transected cells, further indicating the pro-apoptotic role of *ANO6* (TMEM16F) in GIST. Ferroptosis is a new type of non-apoptotic programmed cell death caused by the loss of GPX4 activity and subsequent accumulation of lipid-based ROS and is considered an effective target for cancer treatment [38]. Balachander and Paramasivam identified ferroptosis as an emerging therapeutic target in oral cancer [39]. Delvaux et al. demonstrated that ferroptosis induction and YAP inhibition are new therapeutic targets for GISTs [40]. A previous study found that iron is an important executor of ferroptosis. Intracellular iron levels are regulated by iron regulatory transporters, and Fe^2+^ is particularly important in iron deficiency anemia [41]. Therefore, we explored the effect of *ANO6* (TMEM16F) on ferroptosis in GIST-T1 cells, *in vivo* models, and GIST-T1 IR cells. We found that the *ANO6*-plasmid increased the stimulation of lipid ROS and increased the intracellular concentrations of total iron and Fe^2+^ in GIST-T1 cells, GIST-T1 IR cells, and tissues. We also determined the expression of ferroptosis markers including *Ptgs2* and *Chac1*. Our data revealed that *Ptgs2* and *Chac1* were upregulated in *ANO6*-plasmid transfected cells and tissues compared to the control-plasmid group. The Cys/glutamate reverse transport system Xc^−^ plays an important role in ferroptosis [42]. GSH is one of the most abundant free radical scavengers in cells and one of the most effective regulatory factors of iron deficiency [43]. GPX4 is a central mediator of cancer cell death and is associated with the production of lipid ROS for ferroptosis. Yang et al. revealed the involvement of the GSH-GPX4 pathway in ferroptosis of the retinal pigment epithelium ferroptosis [44]. Moreover, Yang et al. suggested that Maresin1 protects against ferroptosis-induced liver injury through ROS inhibition and Nrf2/HO-1/GPX4 activation [45]. Therefore, we examined the ferroptosis-related pathway regulatory factors, including Cys, GSH, and GPX4. Our findings indicated that the *ANO6*-plasmid reduced Cys, GSH, and GPX4 levels compared to the control-plasmid group.

The Cys/glutamate acid reverse transport system, Xc-, consists of two subunits, *SLC7A11* and *SLC3A2*, which are closely associated with ferroptosis. *SLC7A11* transports Cys into cells, promotes GSH synthesis, and accelerates the inhibition of GPX4 on ferroptosis. Yang et al. have suggested that STAT6 inhibits ferroptosis and alleviates acute lung injury by regulating the P53/*SLC7A11* pathway [26]. In addition, Wu et al. revealed that *SLC3A2* inhibits ferroptosis in laryngeal carcinoma via the mTOR pathway [46]. RT-qPCR and western blotting were used to determine the expression levels of *SLC7A11* and *SLC3A2* in *ANO6*-plasmid transfected GIST-T1 cells. The results suggested that the *ANO6*-plasmid obviously reduced the expression of *SLC7A11* and *SLC3A2* compared to the control-plasmid group. In addition, several studies have shown that pharmacological inhibition of *SLC7A11* plays a vital role in both *in vitro* and *in vivo* models. Based on these findings, we clarified the latent regulatory mechanism of the *ANO6*-plasmid, *SLC7A11*, and *SLC3A2*. Immunohistochemistry assays suggested that the *ANO6*-plasmid prominently increased *ANO6* (TMEM16F) expression and reduced *SLC7A11* and *SLC3A2* expression, demonstrating that *ANO6* (TMEM16F) inhibited GIST growth by regulating *SLC7A11* and *SLC3A2*.

There were also some limitations of this study. First, the specific molecular mechanisms by which *ANO6* (TMEM16F) regulates the biological behavior of GIST cells (involved signaling pathways) still require further analysis and exploration. In addition, the correlation between the expression of *ANO6* (TMEM16F) and pathological parameters in patients with GIST needs to be elucidated. In the future, we will conduct in-depth research on these issues.

## 5 Conclusion

Taken together, our findings provide strong evidence for the mechanisms by which *ANO6* (TMEM16F) induces ferroptosis by regulating *SLC7A11* and *SLC3A2* expression in GIST. This report provides new insights into the treatment of GIST.

## Abbreviations

Cys: cystine
ELISA: enzyme-linked immunosorbent assay
GIST: gastrointestinal stromal tumors
GPX4: glutathione peroxidase 4
GSH: glutathione
IM: imatinib
MTT: 3-(4,5-dimethylthiazol-2-yl)-2,5-diphenyltetrazolium bromide
PBS: phosphate-buffered saline
ROS: reactive oxygen species
RT-qPCR: reverse transcription-quantitative polymerase chain reaction
TUNEL: terminal deoxynucleotidyl transferase dUTP nick-end labeling
